# Efficient diagnosis for endoscopic remission in Crohn’s diseases by the combination of three non-invasive markers

**DOI:** 10.1186/s12876-025-03880-5

**Published:** 2025-05-13

**Authors:** Kensuke Takei, Toshihiro Inokuchi, Sakiko Hiraoka, Mikako Ishiguro, Junki Toyosawa, Yuki Aoyama, Shoko Igawa, Keiko Takeuchi, Yasushi Yamasaki, Hideaki Kinugasa, Masahiro Takahara, Seiji Kawano, Toshiharu Mitsuhashi, Motoyuki Otsuka

**Affiliations:** 1 Department of Gastroenterology and Hepatology, Okayama University Graduate School of Medicine, Dentistry and Pharmaceutical Sciences, 2 - 5- 1 Shikata-Cho, Kita-Ku, Okayama, 700 - 8558 Japan; 2 Research Center for Intestinal Health Science, Okayama University, Okayama, Japan; 3 Center for Innovative Clinical Medicine, Okayama University Hospital, Okayama, Japan

**Keywords:** CD, Crohn’s disease, LRG, Leucine-rich alpha- 2 glycoprotein, Fcal, Fecal calprotectin, CRP, C-reactive protein, ER, Endoscopic remission

## Abstract

**Background:**

Serum C-reactive protein (CRP), leucine-rich alpha-2 glycoprotein (LRG), and fecal calprotectin (Fcal) are non-invasive markers used to assess Crohn’s disease (CD) severity. However, the accuracy of these markers alone is often limited, and most previous reports have evaluated the efficacy of each marker individually. We aimed to improve the diagnostic performance of endoscopic remission (ER) of CD by combining these 3 markers.

**Methods:**

We tested the diagnostic ability of various combinations of these 3 markers for endoscopic severity in 230 consecutive patients with CD from September 2014 to July 2023. The modified Simple Endoscopic Score for Crohn’s disease (mSES-CD) was used to determine endoscopic severity.

**Results:**

Each of the 3 markers was correlated with mSED-CD (LRG: *r* = 0.69, CRP: *r* = 0.60, and Fcal: *r* = 0.67). A combination of 2 of the 3 markers did not increase the diagnostic accuracy of ER. However, by combining all 3 markers, the diagnostic ability for ER was improved in comparison to the diagnostic ability of the 3 individual markers, assuming that ER was obtained if 2 or 3 markers were negative. The sensitivity, specificity, and accuracy were 89%, 83%, and 86%, respectively. Additionally, we established a 2-step method using Fcal values after evaluating the 2 serum markers. This method was most useful for reducing both the patient burden and costs.

**Conclusions:**

The newly established 2-step method allowed for a higher accuracy in the non-invasive diagnosis of ER when the 3 markers were combined.

**Supplementary Information:**

The online version contains supplementary material available at 10.1186/s12876-025-03880-5.

## Introduction

Crohn's disease (CD) is a progressive and intractable inflammatory bowel disease that affects the entire gastrointestinal tract, with small intestinal involvement in 75% of patients [1]. If the inflammation is not properly controlled, irreversible damage accumulates in the intestinal tract, eventually leading to strictures, fistulas, multiple surgeries, and a significant reduction in the patient's quality of life [2, 3]. On the other hand, the emergence of advanced therapies has led to endoscopic remission with a decrease in surgical and admission rates and has brought the treatment to target conception [4, 5]. Precise assessment of the disease status and timely therapeutic intervention can reduce the risk of recurrence [6]. Early detection of flare-ups and appropriate therapeutic intervention to minimize damage to the intestinal tract are paramount in treating CD [5, 7]. In particular, monitoring the disease status of patients with CD, including small intestinal lesions, has become a major clinical concern. While innovative panels like the EHI could be ideal for clinical practice, they are currently unavailable in Japan [2, 8].

Serum C-reactive protein (CRP) and fecal calprotectin (Fcal) have been reported to be useful surrogate markers of bowel inflammation in patients with CD [8–12]. In particular, Fcal shows a high correlation with endoscopic severity and can non-invasively diagnose mucosal healing with high accuracy. Leucine-rich alpha- 2 glycoprotein (LRG) is a novel serum marker for inflammatory bowel disease (IBD) and has been recently reported to be more accurate than CRP as a marker of endoscopic severity and might be equivalent to Fcal [13–16]. However, previous reports have been limited to evaluating the efficacy of individual markers or the combination of 2 markers, and the ability of these markers for diagnosing endoscopic remission was not satisfactory. Because there have been no attempts to evaluate disease activity using 3 markers for monitoring patients with CD, we aimed to determine the efficacy of the diagnostic performance by combining 3 markers: CRP, LRG, and Fcal.

## Materials and methods

### Patients

Consecutive CD patients who underwent endoscopy at Okayama University Hospital between September 2014 and July 2023 and whose stool and blood samples were scheduled to be collected were considered eligible for this study. All patients had undergone previous endoscopic examinations with findings of active inflammation in the colon, in the small bowel, or in both. CD patients with colonic lesions alone underwent colonoscopy, while CD patients with lesions in the small bowel underwent balloon-assisted enteroscopy (BAE). All patients provided blood samples for the determination of serum LRG and CRP levels on the morning of the day when endoscopy was performed. In addition, all patients were instructed to collect stool samples at home within 2 days before endoscopy and bring them to the hospital on the day of endoscopy to test the Fcal values. The patients’ clinical characteristics, including age at the diagnosis, sex, disease location, disease behavior, and current medications, were obtained. The clinical disease activity for patients with CD was evaluated using the Crohn’s disease activity index (CDAI), with clinical remission defined as CDAI < 150.

The exclusion criteria were insufficient stool collection, having had a colostomy or ileostomy, and failure to achieve full endoscopic observation of the lesions. In addition, patients with background factors that could affect the levels of LRG, CRP, and Fcal, including uncontrolled perianal disease, extraintestinal complications, collagen disease, heart failure, infectious disease, immediate postvaccination, and malignancy at the time of endoscopy, were excluded.

### Endoscopy procedures and the assessment of endoscopic disease activity

According to our hospital’s standard protocol, colonoscopy or BAE was performed after bowel preparation with either a polyethylene glycol-based or magnesium citrate-based electrolyte solution. A double-balloon enteroscope EN- 580 T (Fujifilm, Tokyo, Japan) was used by experienced endoscopists (T.I. and S.K.). The DBE scope was inserted into the proximal small intestine as far as possible using a retrograde approach. Complete observation of the affected lesions was accomplished in all patients, and those in whom the affected lesions could not be fully evaluated (e.g., due to stenosis) were excluded.

Endoscopic assessment was scored using the modified Simple Endoscopic Score for Crohn’s Disease (mSES-CD), which was based on the original SES-CD and evaluated 3 items (ulcer size, ulcer area, and lesion area) in 6 segments (proximal and distal parts of the small intestine, right colon, transverse colon, left colon, and rectum) [10, 11, 17]. The mSES-CD score was able to reflect the small bowel activity in more detail by dividing the ileum into 2 segments: distal and proximal ileum. The mSES-CD score defined the distal ileum as the part of the ileum within 40 cm of the ileocecal valve or anastomosis, whereas the score defined the proximal ileum as the deeper part of the ileum ≥ 40 cm proximal to those points [17]. Endoscopic remission was defined by the mSES-CD score of 0 to 2, according to SES-CD [17–20]. Endoscopic findings were evaluated with the endoscopist blinded to the results of serum and fecal markers.

### The measurement of serum LRG levels

Blood samples were collected and serum LRG levels were measured using a NANOPIA LRG (SEKISUI MEDICAL Company Limited, Tokyo, Japan) in an in-hospital laboratory. SEKISUI MEDICAL Company Limited provided the measuring reagents and funded a portion of the LRG-measuring reagent cost. The quantitative range for LRG was 5.0 to 100 µg/mL.

### Fecal calprotectin analysis

The level of calprotectin were measured by a fluorescence enzyme immunoassay using Phadia EliA™ Calprotectin 2 (Thermo Fisher Scientific, Phadia AB, Uppsala, Sweden) in fecal samples sent to BML (Tokyo, Japan). The quantitative range for calprotectin was 0.65 to 84,000 µg/g, with fecal samples diluted appropriately from 1:50 to 1:100,000.

### Patient prognosis

The relationship between the results of 3 markers and patient prognosis, including relapse rate, CD-related hospitalization rate, and CD-related surgery rate, was evaluated during observation periods. Relapse was defined as a requirement for a change in treatment (i.e., the use of corticosteroids, the administration of a new biologic therapy, or a change in biologic therapy).

### Statistical analyses

All statistical analyses were conducted using the JMP software program (version 16.0 pro for Windows, SAS Institute Inc., Cary, NC, USA). The Shapiro–Wilk test was used to determine whether the distribution of continuous variables was normal. Variables with a non-normal distribution were summarized as the median (interquartile range [IQR]), and group comparisons were performed using the Mann–Whitney U test. Pearson’s correlation coefficient or Spearman’s correlation coefficient was used to analyze the association between the serum, fecal marker, disease activity, and other biomarkers. Receiver operating characteristic (ROC) curve analysis was performed using a logistic regression model, and DeLong’s test was employed to compare two correlated ROC curves. The cut-off values were determined using the Youden’s index. The results are expressed as the area under the curve (AUC) with sensitivity, specificity, positive predictive value (PPV), negative predictive value (NPV), and accuracy, with 95% confidence intervals (CI). A k-fold cross-validation was adopted to validate the diagnostic ability of multiple markers. Regarding k-fold cross-validation, we performed cross-validation from twofold to tenfold on each subject's data and adopted the k value with the highest diagnostic ability [21–23]. AUC was calculated using k-fold cross-validation, and the results were compared across different marker combinations. Analyses of relapse, hospitalization, and colectomy rates were carried out using the Kaplan and Meier method. Statistical comparison was carried out by Log-rank test. Univariate analysis using a Cox proportional hazard model was also conducted to evaluate the risks for prognosis. All *P*-values were 2-sided, and *P*-values < 0.05 were considered statistically significant. Data underlying this article are available from the corresponding author upon reasonable request.

### Ethical statements

This study protocol, conducted in accordance with the Declaration of Helsinki, was reviewed and approved by the Institutional Review Board of the Okayama University Graduate School of Medicine (IRB: 1904–035). Written informed consent was obtained from all participants involved in this study.

## Results

### Clinical characteristics of the enrolled patients

Among 268 consecutive outpatients with CD enrolled in this study, 38 patients, whose Fcal values could not be measured due to insufficient stool specimens were excluded. The remaining 230 patients (149 males and 81 females; median age at diagnosis, 24 years) were included in this study. These patients underwent endoscopy and their serum and fecal markers were examined simultaneously. The clinical characteristics of the patients and their values are summarized in Table 1. Regarding the disease locations of the 230 cases, 50 (22%) had ileal lesions alone, 47 (20%) had colonic lesions alone, and 133 (58%) had ileocolonic lesions. All patients with ileal lesions underwent BAE, regardless of the presence or absence of lesions proximal to the terminal ileum. A tumor necrosis factor-alpha antagonist was administered to 125 patients (54%) and thiopurine was administered to 98 patients (43%). Of the 230 patients, 155 (67%) were in clinical remission, whereas 75 (33%) had clinically active disease. The endoscopic findings were as follows: mSES-CD 0, *n* = 97 (42%); mSES-CD 1–2, *n* = 19 (8%); mSES-CD 3–4, *n* = 54 (24%); mSES-CD 5–10, *n* = 57 (25%); and mSES-CD 11–15, *n* = 2 (1%). A normality test for continuous variables, including CRP, LRG, and Fcal, was performed using the Shapiro–Wilk test, revealing a non-normal distribution for all markers (*P* < 0.0001). The median (IQR) values of these biomarkers were 13.1 (8.5–16.7) μg/mL for LRG, 0.10 (0.04–0.22) mg/dL for CRP, and 202 (61–687) μg/g for Fcal.

**Table 1:** Characteristics of the patients enrolled in this study

Patients	
Total	*n* = 230 (%)
Gender	
Male/Female	149 (65)/81 (35)
Median (IQR) age of undergoing endoscopy (years)	44 (34–57)
Median (IQR) duration of disease (years)	12 (4.4–21)
Median (IQR) age at diagnosis (years)	24 (19–34)
A1: < 16/A2:16–40/A3: > 40	36 (16)/166 (72)/28 (12)
Number of endoscopy procedures	
1/2/> 3	84 (59)/40 (28)/19 (13)
Disease location	
L1: ileal/L2: colonic/L3: ileocolonic	50 (22)/47 (20)/133 (58)
Disease behavior	
B1: inflammation/B2: stricturing/B3: penetrating	75 (33)/94 (41)/61 (26)
Perianal disease	98 (43)
CDAI score (IQR)	135 (58–179)
Clinical remission^a^	155 (67)
Previous intestinal resection	130 (57)
Smoking (current/ex/never)	12 (5)/25 (11)/193 (84)
Concomitant medications	
5-aminosalicylic acid	153 (67)
Corticosteroids	31 (13)
Thiopurine	98 (43)
TNF-alpha antagonist	125 (54)
Vedolizumab	1 (0.4)
Ustekinumab	32 (14)
Elemental diet	128 (56)
**Endoscopy**	
Total colonoscopy/Balloon-assisted enteroscopy	47 (20)/183 (80)
Endoscopy findings	
mSES-CD 0/1–2/3–4/5–10/11–15	97 (42)/19 (8)/54 (24)/57 (25)/3 (1)
endoscopic remission (≤ 2)	116 (50%)
**Values of biomarkers, median (IQR)**	
Leucine-rich alpha- 2 glycoprotein (µg/mL)	13.1 (8.5–16.7)
C-reactive protein (mg/dL)	0.10 (0.04–0.22)
Fecal calprotectin (μg/g)	202 (61–687)

*IQR* interquartile range, *mSES-CD* modified simple endoscopic score for Crohn’s disease, *CDAI* Crohn’s disease activity index, *TNF* tumor necrosis factor

^a^Clinical remission was defined as a CDAI score of < 150

### Correlations between LRG/CRP/Fcal values, CDAI and mSES-CD in patients with CD

**Fig. 1:**
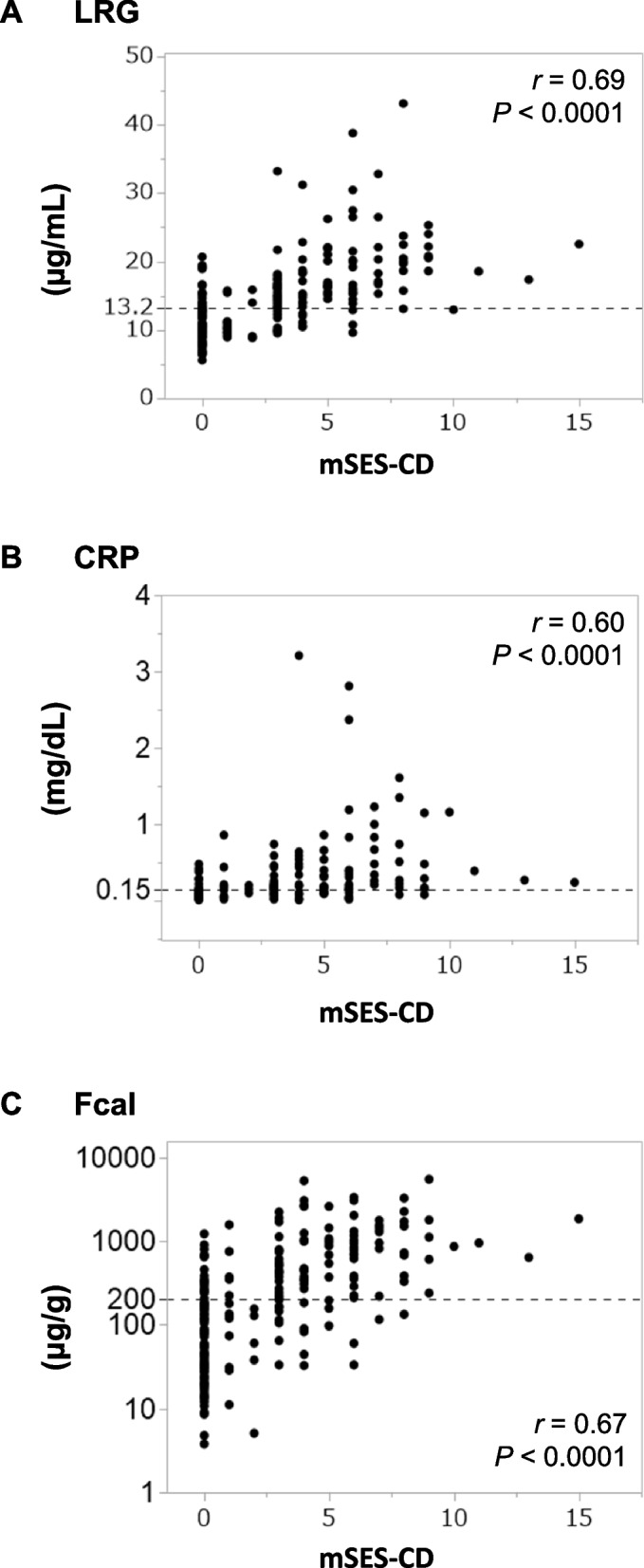
Correlation between the values of the single use of non-invasive markers and the mSES-CD. The values of each marker (**A**, LRG, **B**, CRP, and **C**, Fcal) and mSES-CD in the enrolled cases. LRG, leucine-rich alpha- 2 glycoprotein; CRP, C-reactive protein; Fcal, fecal calprotectin; mSES-CD, modified simple endoscopic score for Crohn’s disease. Parallel dotted lines show the cut-off values of the non-invasive markers used in this study. The cut-off values were as follows: LRG, 13.2 µg/mL; CRP, 0.15 mg/dL; and Fcal, 180 µg/g

Correlations between the mSES-CD and the values of each marker (LRG, CRP, and Fcal) were analyzed. Spearman’s rank correlation coefficients and *p*-values were as follows: LRG: *r* = 0.69, *P* < 0.0001; CRP: *r* = 0.60, *P* < 0.0001; and Fcal: *r* = 0.67, *P* < 0.0001 (Fig. 1). These results indicated that all 3 markers are significantly correlated with mSES-CD and that LRG and Fcal show the highest correlations when limited to the cases with ileal lesions (*r* = 0.63) and limited to the cases with ileocolonic lesions *(r* = 0.73, respectively (Supplementary Fig. 1–3). On the other hand, there were low correlations between each marker, mSES-CD and CDAI (LRG: *r* = 0.23, *P* < 0.001; CRP: *r* = 0.08, *P* = 0.24; and Fcal: *r* = 0.21, *P* < 0.005, Supplementary Fig. 4).

**Fig. 2:**
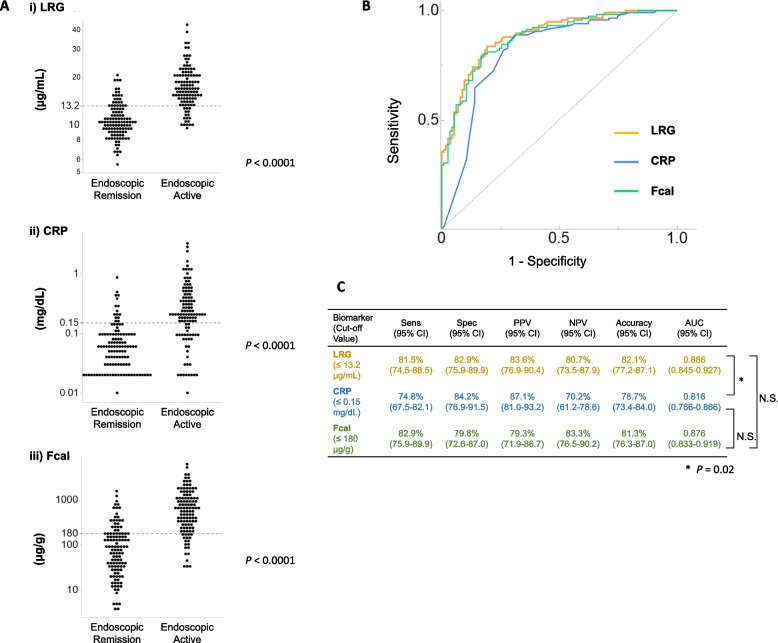
Diagnostic ability by the single use of the individual non-invasive markers for endoscopic remission in CD. **A** The values of each marker were significantly decreased in the group with endoscopic remission. Parallel dotted lines show the cutoff values of the serum/fecal biomarkers in this study: LRG, 13.2 µg/mL; CRP, 0.15 mg/dL; and Fcal, 180 µg/g.** B** The diagnostic ability of the single use of each marker for endoscopic remission. **C** The receiver operating characteristic curve for the diagnosis of endoscopic remission by a single use of each marker. LRG, leucine-rich alpha- 2 glycoprotein; CRP, C-reactive protein; Fcal, fecal calprotectin; Sens, sensitivity; Spec, specificity; PPV, positive predictive value; NPV, negative predictive value; AUC, area under the curve; CI, confidence interval

Next, the abilities of each marker for diagnosing endoscopic remission were assessed individually. When comparing the cases with endoscopic remission and the cases with endoscopically active lesions, the values of each marker were significantly lower in the endoscopic remission group than in the “endoscopic active” group (Fig. 2A; LRG, *P* < 0.0001; CRP, *P* < 0.0001; and Fcal, *P* < 0.0001). The AUC values for endoscopic remission were as follows: LRG, 0.886 (95% confidence interval (CI), 0.845–0.927); CRP, 0.816 (95% CI, 0.746–0.866); Fcal, 0.876 (95% CI, 0.833–0.919) and CDAI, 0.614 (95% CI, 0.573–0.652). The optimal cut-off levels of each marker, calculated using the Youden index to diagnose endoscopic remission non-invasively, were as follows: LRG, 13.2 μg/mL; CRP, 0.15 mg/dL and Fcal, 180 μg/g (Fig. 2B and C). Only LRG and CRP showed statistically significant differences in AUC values (*P* = 0.02).

### A combinatorial method using 2 markers to diagnose endoscopic remission in patients with CD

When evaluating the diagnostic ability of the combination using only 2 of the 3 markers, the accuracy was not superior to any of the individual markers, regardless of the combination (Table 2; upper part). Among these, the combination of CRP and LRG had the best ability for the diagnosis of endoscopic remission. The sensitivity, specificity, PPV, NPV, accuracy rate, and AUC were 74.2%, 90.0%, 89.0%, 76.2%, 81.7%, and 0.831, respectively).

**Table 2:** Diagnostic performance of the combination of CRP, Fcal and LRG for endoscopic remission

	Sensitivity (95% CI)	Specificity (95% CI)	PPV (95% CI)	NPV (95% CI)	Accuracy (95% CI)	AUC (95% CI)
**Combination of 2 markers**						
CRP (-) and LRG (-)	74.2% (66.3–82.0)	90.0% (84.4–95.6)	89.0% (82.9–95.1)	76.2% (68.8–83.5)	81.7% (76.7–86.7)	0.831 (0.7823–0.879)
CRP (-) and Fcal (-)	69.0% (60.5–77.4)	93.9% (89.5–98.3)	92.0% (86.2–97.7)	74.8% (67.7–81.9)	81.3% (76.3–86.3)	0.814 (0.764–0.864)
Fcal (-) and LRG (-)	65.5% (56.9–74.2)	97.4% (94.4–1.00)	96.2% (92.0–1.00)	73.5% (66.5–80.5)	81.3% (76.3–86.3)	0.814 (0.764–0.864)
**Combination of 3 markers**						
Two or more markers (-) of the 3 markers	89.4% (83.5–95.3)	83.3% (76.8–89.8)	81.6% (74.5–88.7)	90.5% (85.2–95.8)	86.1% (81.6–90.6)	0.860 (0.816–0.905)
CRP (-), Fcal (-), and LRG (-)	71.3% (64.2–78.3)	100% (100–100)	100% (100–100)	60.3% (51.4–69.2)	80.0% (74.8–85.1)	0.794 (0.742–0.846)

*AUC* area under the curve, *PPV* positive predictive value, *NPV* negative predictive value, *LRG* leucine-rich alpha- 2 glycoprotein, *CRP* C-reactive protein, *Fcal* fecal calprotectin, *CI* confidence interval

**Table 3:** Validation of the predictive diagnostic ability of the 3 markers supported by k-fold cross-validation

	Sensitivity (95% CI)	Specificity(95% CI)	PPV (95% CI)	NPV (95% CI)	Accuracy (95% CI)	AUC (95% CI)	*P*-value (95% CI)
The 3-marker method (two or more markers (-)of the 3 markers)	89.7 (82.6–94.5)	80.7% (72.3–87.5)	82.5% (74.8–88.7)	88.5% (80.7–93.9)	85.2% (80.0–89.5)	0.852 (0.806–0.898)	
All three markers negative (CRP (-), Fcal (-), and LRG (-))	56.9 (47.4–66.1)	100.0% (96.8–100.0)	100.0% (94.6–100.0)	69.5% (61.9–76.5)	78.3% (72.4–83.4)	0.785 (0.739–0.830)	0.0190
CRP (-) and Fcal (-)	67.2 (57.9–75.7)	93.9% (87.8–97.5)	91.8% (83.8–96.6)	73.8% (65.8–80.7)	80.4% (74.7–85.4)	0.806 (0.757–0.854)	0.0654
Fcal (-) and LRG (-)	63.8 (54.4–72.5)	96.5% (91.3–99.0)	94.9% (87.4–98.6)	72.4% (64.5–79.3)	80.0% (74.2–85.0)	0.801 (0.754–0.849)	0.0589
CRP (-) and LRG (-)	72.4 (63.3–80.3)	90.4% (83.4–95.1)	88.4% (80.2–94.1)	76.3% (68.2–83.2)	81.3% (75.7–86.1)	0.814 (0.765–0.863)	0.0906

*AUC* area under the curve, *PPV* positive predictive value, *NPV* negative predictive value, *LRG* leucine-rich alpha- 2 glycoprotein, *CRP* C-reactive protein, *Fcal* fecal calprotectin, *CI* confidence interval

### A combination method using all 3 markers to diagnose endoscopic remission in patients with CD

Next, all 3 markers (CRP, LRG, and Fcal) were simultaneously used to diagnose the endoscopic activity. In this case, “endoscopic remission” was defined when 2 or more markers were negative, and “endoscopic active” disease was defined when 2 or more markers were positive. The diagnostic ability for endoscopic remission was improved in comparison to individual markers or the combination of any 2 markers. The sensitivity, specificity, PPV, NPV, and accuracy rates were 89.4%, 83.3%, 81.6%, 90.5%, and 86.1%, respectively (Table 2; lower part). Furthermore, if endoscopic remission was defined as all 3 negative markers with negative, the sensitivity, specificity, PPV, NPV, and accuracy in the diagnosis of endoscopic remission were 71.3%, 100%, 100%, 60.3%, and 80.0%, respectively.

### Validation of the diagnostic ability of the combination method using 3 markers in patients with CD

**Fig. 3:**
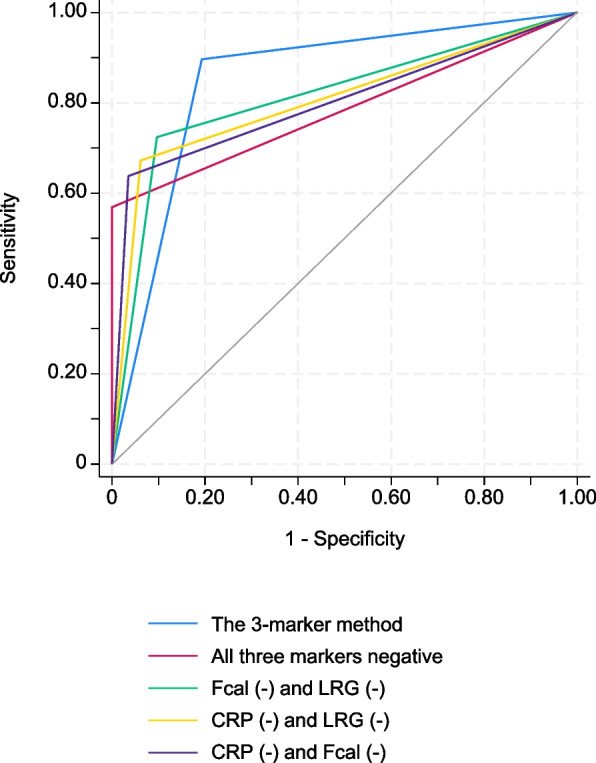
The 3-marker method is superior to other combination methods using any 2 of the 3 markers. The receiver operating characteristic curves for monitoring endoscopic remission using combinations of 2 or 3 markers. The results of the comparison of AUCs using k-fold cross-validation are shown. LRG, leucine-rich alpha- 2 glycoprotein; CRP, C-reactive protein; Fcal, fecal calprotectin; AUC, area under the curve

To validate the diagnostic ability of the combination method using the 3 markers, we first performed k-fold cross-validation by comparing it with other combinations using CRP, LRG, and Fcal (Table 3). The combination method using 3 markers was superior in terms of sensitivity, specificity, and accuracy compared to other combination methods when the negativity of 2 or more markers corresponded to endoscopic remission. Next, we compared the diagnostic abilities of the combination method using 3 markers and those of the existing 2-marker methods using k-fold cross-validation (Fig. 3). In these analyses, the 3-marker method, which defined endoscopic remission as negativity in two or more markers, had the highest AUC value (AUC: 0.852) and tended to be superior to other two-marker methods; however, the difference was not statistically significant. The AUC values for the other methods were as follows: CRP (-) and Fcal (-): 0.806 (*P* = 0.0654), Fcal (-) and LRG (-): 0.801 (*P* = 0.0589), and CRP (-) and LRG (-): 0.814 (*P* = 0.0906).

### The long-term prognosis of the patients with CD

**Fig. 4:**
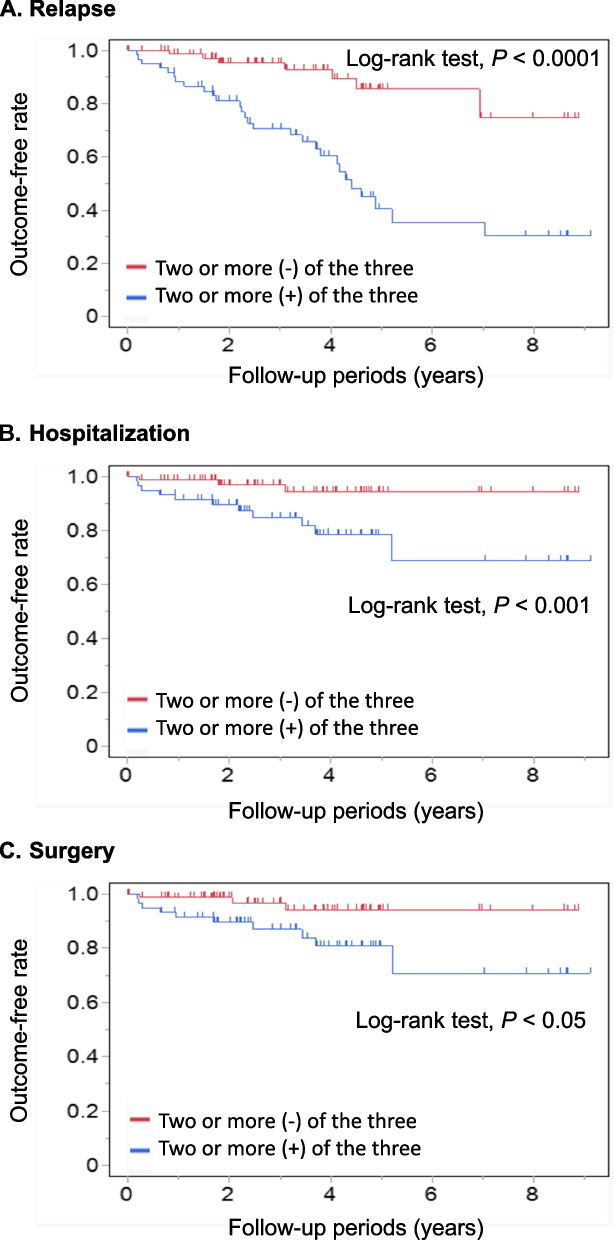
Kaplan–Meier curves for the rate of relapse, hospitalization, and surgery stratified with the results of 3 markers. **A** Relapse (**B**) Hospitalization (**C**) Surgery. Patients with two or more negative markers had a more favorable prognosis, including a reduced risk of relapse, hospitalization, and surgery (*P* < 0.0001, *P* < 0.001, and *P* < 0.05, respectively)

During the median observation period of 3.6 years (IQR; 1.8–4.7), 35 patients had a relapse requiring a change in treatment, 14 were hospitalized, and 13 underwent surgery due to the disease course of CD. Kaplan–Meier analysis showed that Patients with two or more negative of the three markers had a more favorable prognosis in terms of reduced risk of relapse, with a hazard ratio (HR) of 0.20 (95% CI: 0.09–0.45, *P* < 0.0001) (Fig. 4A). Similarly, patients with two or more negative markers were significantly more likely to avoid hospitalization (HR: 0.21 (95% CI: 0.06–0.77, *P* < 0.001) and surgery (HR: 0.24 (95% CI: 0.07–0.88), *P* < 0.05) (Fig. 4B and C).

### A 2-step method using 3 markers is the best diagnostic method for endoscopic remission in patients with CD

**Fig. 5:**
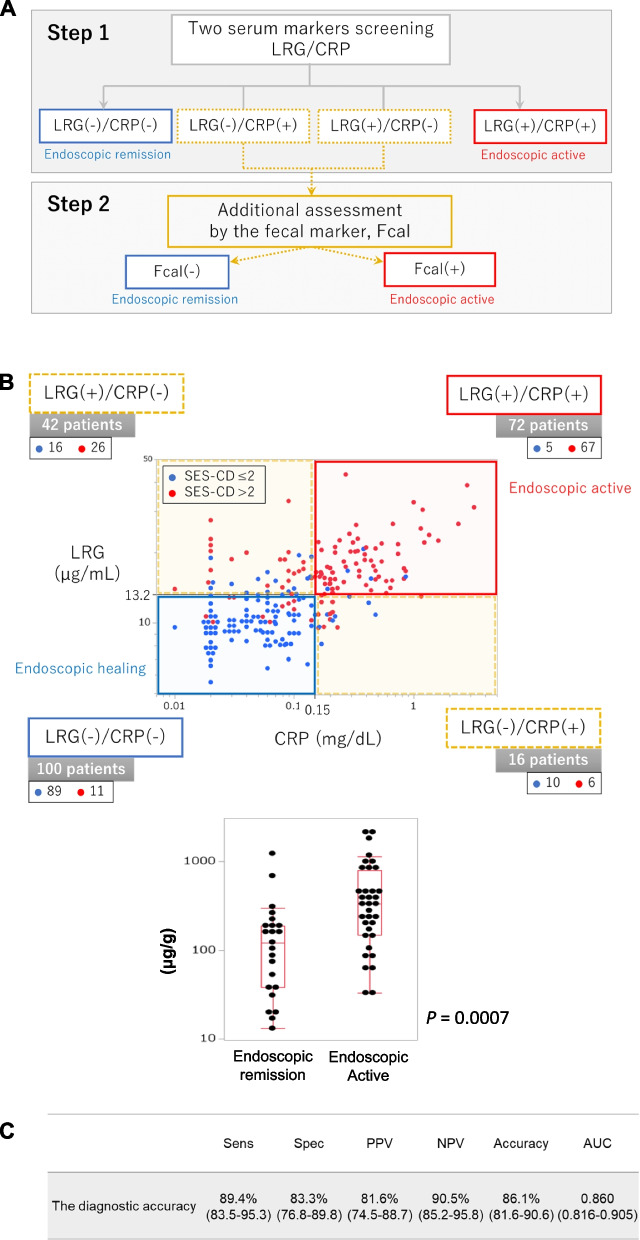
The “2-step method” using 3 markers showed the best diagnostic ability for monitoring endoscopic remission in CD. **A** The flow of the “2-step method” for monitoring endoscopic remission in CD patients. **B** A scatter plot using the CRP and LRG results and additional evaluation by Fcal in the discordant groups. **C** The diagnostic ability of the 2-step method for monitoring endoscopic remission in patients with CD. LRG, leucine-rich alpha- 2 glycoprotein; CRP, C-reactive protein; Fcal, fecal calprotectin; Sens, sensitivity; Spec, specificity; PPV, positive predictive value; NPV, negative predictive value

While LRG and CRP are convenient as serum markers, Fcal, a fecal marker, has some inconvenience because it requires stool and sample collection for testing. Therefore, from a practical perspective, we next examined the diagnostic ability of a “2-step method” using the markers for the diagnosis of endoscopic remission (Fig. 5A). To establish the 2-step method, we first drew a scatterplot based on the values of serum markers, CRP, and LRG. Then, the patients were divided into four groups using the optimal cut-off values. The 2 groups with double-positive (CRP [+] and LRG [+]) or double-negative (CRP [-] and LRG [-]) were defined as “endoscopic remission” or “endoscopic active” (blue and red groups) (Fig. 5B). For other groups that showed positivity for CRP or LRG (yellow groups), Fcal was measured as the second step. We determined whether the case was in “endoscopic remission” or “endoscopic active” based on the Fcal results. Using this “2-step method”, the sensitivity, specificity, PPV, NPV, and accuracy rate for endoscopic remission were 89.4%, 83.3%, 81.6%, 90.5%, and 86.1% (Fig. 5C). As a result, this 2-step method had the highest diagnostic ability among the measurement methods while minimizing the burden of the measurement of Fcal, which may be beneficial for the patient and from the viewpoint of medical economics.

## Discussion

The non-invasive diagnosis of the endoscopic lesions is crucial for monitoring patients with CD. This study demonstrated that the diagnostic accuracy for the non-invasive diagnosis of endoscopic remission was improved by the use of 3 markers: CRP, LRG, and Fcal. In particular, the 3-marker simultaneous measurement method was more effective than the single use of any individual marker or the 2-marker combination method and could predict the prognosis regarding relapse, hospitalization, and surgery. Moreover, instead of testing 3 markers simultaneously, it was possible by introducing the “2-step method” to reduce the number of Fcal measurements, which requires time and effort, without sacrificing the diagnostic accuracy.

CD irreversibly causes inflammatory damage to the intestinal tract, and close and continuous monitoring is essential to prevent intestinal complications and implement a treat-to-target strategy [6]. In particular, approximately 80% of patients with CD have small intestinal lesions, and it is necessary to not only evaluate the terminal ileum but also perform a close examination of the small intestine using a small intestine endoscope [1]. However, endoscopic follow-up of the intestinal tract, including the small intestine, is highly invasive, making it difficult to perform frequent small intestine endoscopy, and less invasive and less costly markers are recommended for monitoring. The evidence of CRP and Fcal has been well established for monitoring disease activity in patients with CD. Additionally, based on recent reports, the accuracy rate of LRG alone for diagnosing endoscopic remission was estimated to be approximately 80%, which is more useful than CRP and has been reported to be at the same level as Fcal [14, 15]. However, there have been no reports on the 3 types of combinations of markers, and this is the first report on the potential of the combination of LRG with CRP and Fcal to improve the accuracy rate for diagnosing endoscopic remission. A statistical validation method (k-fold cross-validation) was employed to assess the diagnostic ability of endoscopic remission using a combination of 3 distinct markers and it was demonstrated that the number of positive markers among the three significantly predicted the long-term prognosis, including relapse rate, hospitalization rate, and surgery rate. Furthermore, the “2-step method” would be beneficial in determining whether a certain number of patients were in endoscopic remission based solely on blood markers, eliminating the inconvenience of treating stool samples. In fact, 38 patients whose Fcal levels could not be measured owing to insufficient stool specimens were excluded from this study.

The present study was associated with several limitations. First, the study was conducted at a single hospital, which may introduce bias, as the patient group may be dominated by those with mild disease activities. Approximately 70% of the patients in this study were treated with biologics, possibly because of their generally mild endoscopic severity. To demonstrate the reproducibility and universality of the results, studies should be conducted at multiple institutions. Further verification of the cut-off values of three markers for the diagnosis of endoscopic remission is necessary. Although previous reports commonly use a cut-off value of, because there are various evaluation methods for small intestinal lesions in CD, more investigation is required [14–16]. In addition, the cut-off values for CRP and Fcal in this study were lower than those recommended by the AGA (Supplementary Table 1). Specifically, CRP was 1.5 mg/L and Fcal was 180 µg/g, whereas the AGA-recommended values were 5 ± 5 mg/L for CRP and 250 ± 50 µg/g for Fcal. This difference may be due to the use of mSES-CD scoring with BAE in CD patients with small intestinal lesions, allowing detection of subtle inflammation that might be missed by colonoscopy alone. As a result, our cut-off values were slightly lower than those based on SES-CD, which relies on colonoscopy. A previous study using BAE reported cut-off values of 0.1–0.5 mg/L for CRP and 77–254 µg/g for Fcal, which are generally consistent with our findings [24–27]. However, the discrepancy between AGA-recommended cut-offs and those derived from multiple BAE-based studies remains insufficiently explored, warranting further investigation. Third, no comparisons were made with other novel biomarker panels, such as the EHI panel proposed in previous studies [28]. Both CRP and Fcal, widely used markers for CD with extensive global evidence, are advantageous in terms of simplicity and cost-effectiveness but demonstrate suboptimal diagnostic performance [29]. In this study, we investigated whether adding LRG, a novel marker recently introduced in Japan, could further enhance diagnostic performance. Lastly, currently in Japan, the medical insurance system does not allow simultaneous measurement of all 3 markers. In light of these circumstances, the implementation of a 2-step methodology allows for the identification of high-risk cases, followed by additional fecal calprotectin testing in cases suspected of being at risk in actual clinical practice. This approach offers the potential for more cost-effective and patient-centered care.

In conclusion, our study demonstrated that combining CRP, Fcal, and LRG can be a non-invasive diagnostic tool that can monitor endoscopic remission with greater accuracy and long-term prognosis. Further research is necessary to determine more appropriate cut-off values and to identify more effective ways of utilizing these markers.

## Supplementary Information

Supplementary Material 1.

Supplementary Material 2.

## Data Availability

The data supporting the findings of this study are available on reasonable request to the corresponding author.

## Abbreviations

CD: Crohn’s disease
ER: Endoscopic remission
CRP: C-reactive protein
Fcal: Fecal calprotectin
LRG: Leucine-rich alpha-2 glycoprotein
BAE: Balloon-associated enteroscopy
CDAI: Crohn’s disease activity index
SES-CD: Simple endoscopic score for Crohn’s disease
ROC: Receiver operating characteristics curve
AUC: Area under the curve
PPV: Positive predictive value
NPV: Negative predictive value
