# Supplementary material for: Efficient diagnosis for endoscopic remission in Crohn’s diseases by the combination of three non-invasive markers
Source: BMC Gastroenterol. 2025 May 13;25:364. doi: 10.1186/s12876-025-03880-5 (PMC12070669; doi:10.1186/s12876-025-03880-5)
Supplement: Supplementary file 1 — Supplementary Material 1. [file 12876_2025_3880_MOESM1_ESM.pptx]

## Slide 1
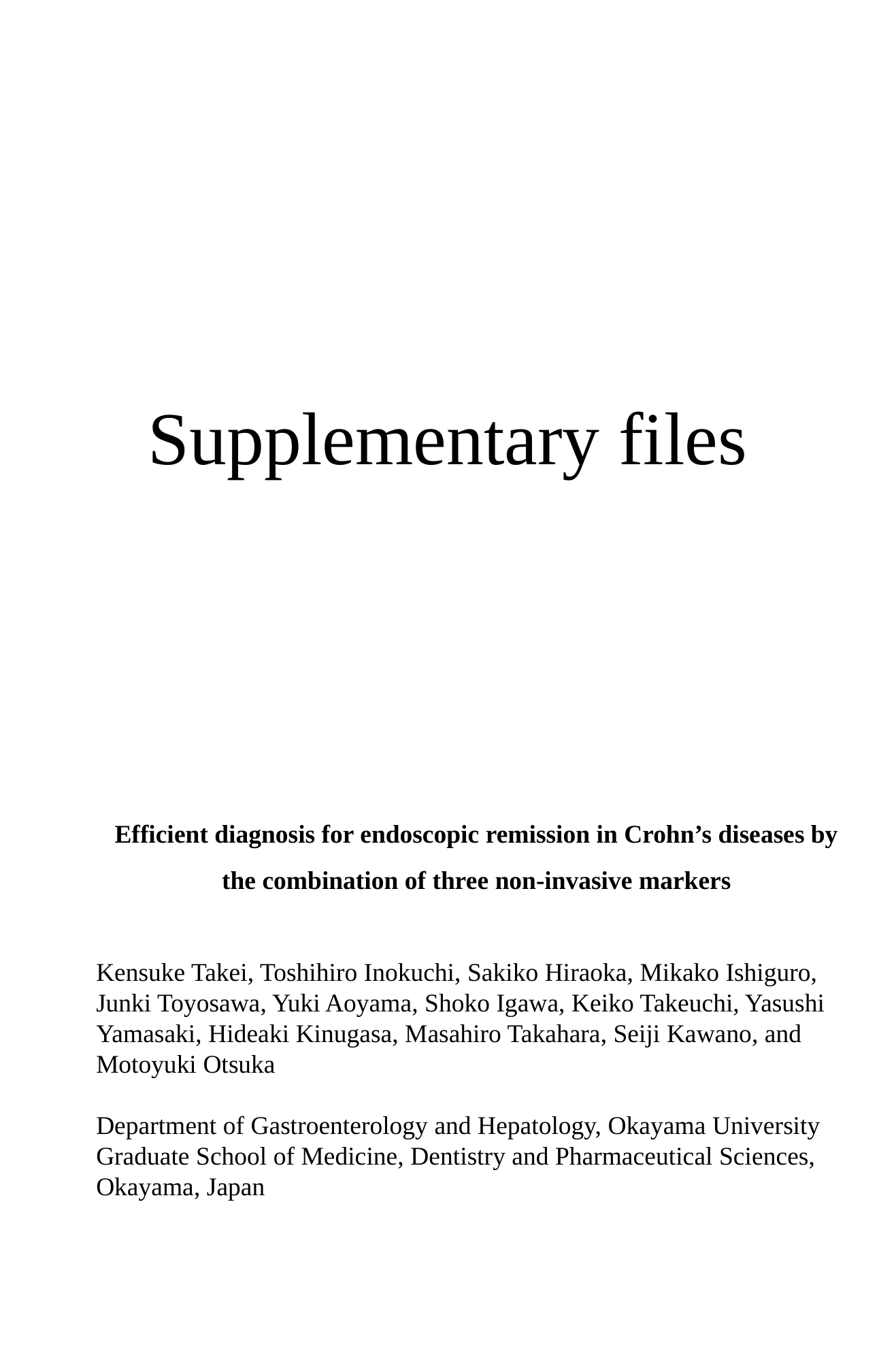

# Supplementary files
Efficient diagnosis for endoscopic remission in Crohn’s diseases by the combination of three non-invasive markers
Kensuke Takei, Toshihiro Inokuchi, Sakiko Hiraoka, Mikako Ishiguro, Junki Toyosawa, Yuki Aoyama, Shoko Igawa, Keiko Takeuchi, Yasushi Yamasaki, Hideaki Kinugasa, Masahiro Takahara, Seiji Kawano, and Motoyuki Otsuka
Department of Gastroenterology and Hepatology, Okayama University Graduate School of Medicine, Dentistry and Pharmaceutical Sciences, Okayama, Japan

## Slide 2
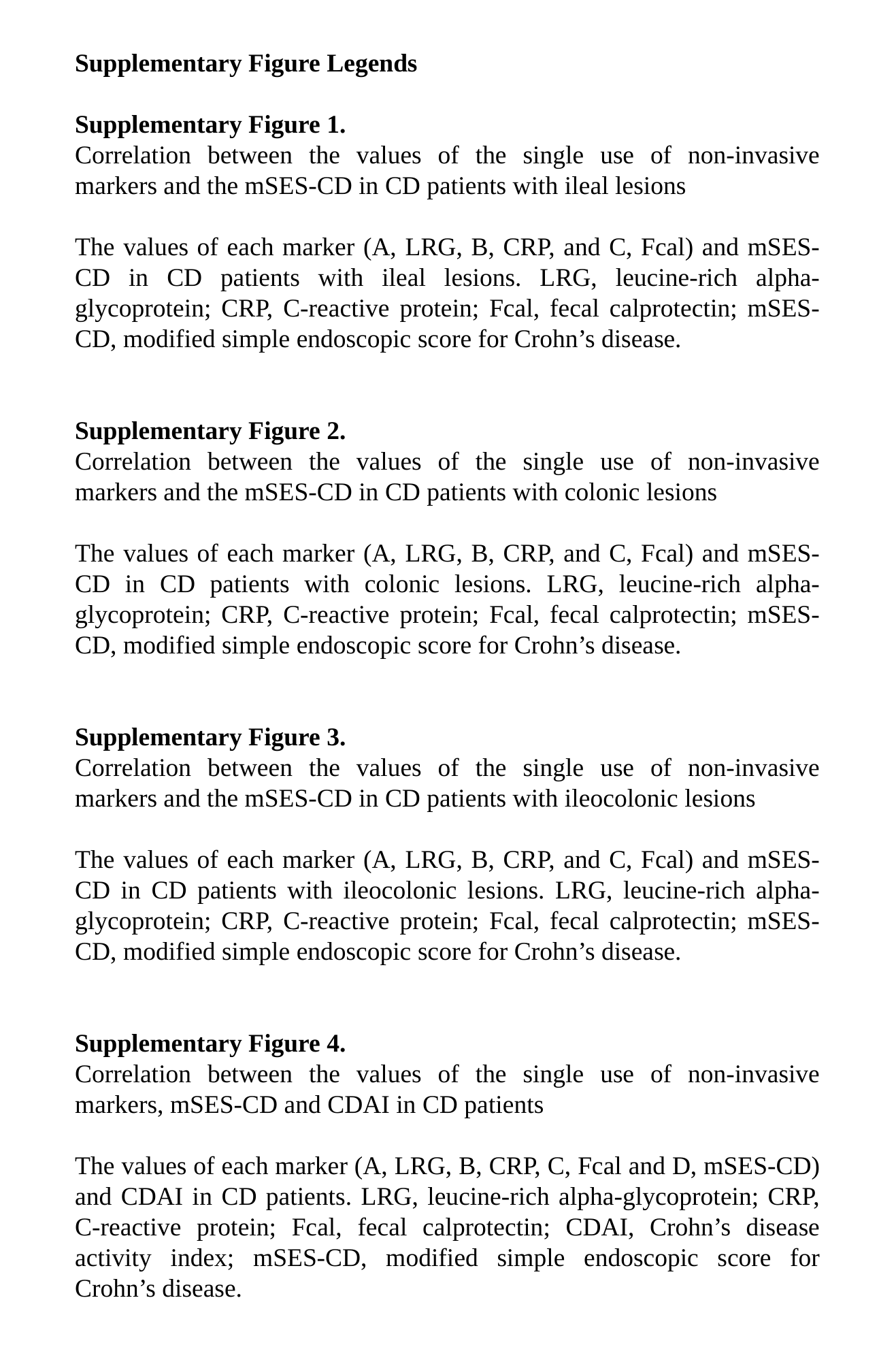

Supplementary Figure Legends
Supplementary Figure 1.
Correlation between the values of the single use of non-invasive markers and the mSES-CD in CD patients with ileal lesions
The values of each marker (A, LRG, B, CRP, and C, Fcal) and mSES-CD in CD patients with ileal lesions. LRG, leucine-rich alpha- glycoprotein; CRP, C-reactive protein; Fcal, fecal calprotectin; mSES-CD, modified simple endoscopic score for Crohn’s disease.
Supplementary Figure 2.
Correlation between the values of the single use of non-invasive markers and the mSES-CD in CD patients with colonic lesions
The values of each marker (A, LRG, B, CRP, and C, Fcal) and mSES-CD in CD patients with colonic lesions. LRG, leucine-rich alpha- glycoprotein; CRP, C-reactive protein; Fcal, fecal calprotectin; mSES-CD, modified simple endoscopic score for Crohn’s disease.
Supplementary Figure 3.
Correlation between the values of the single use of non-invasive markers and the mSES-CD in CD patients with ileocolonic lesions
The values of each marker (A, LRG, B, CRP, and C, Fcal) and mSES-CD in CD patients with ileocolonic lesions. LRG, leucine-rich alpha- glycoprotein; CRP, C-reactive protein; Fcal, fecal calprotectin; mSES-CD, modified simple endoscopic score for Crohn’s disease.
Supplementary Figure 4.
Correlation between the values of the single use of non-invasive markers, mSES-CD and CDAI in CD patients
The values of each marker (A, LRG, B, CRP, C, Fcal and D, mSES-CD) and CDAI in CD patients. LRG, leucine-rich alpha-glycoprotein; CRP, C-reactive protein; Fcal, fecal calprotectin; CDAI, Crohn’s disease activity index; mSES-CD, modified simple endoscopic score for Crohn’s disease.

## Slide 3
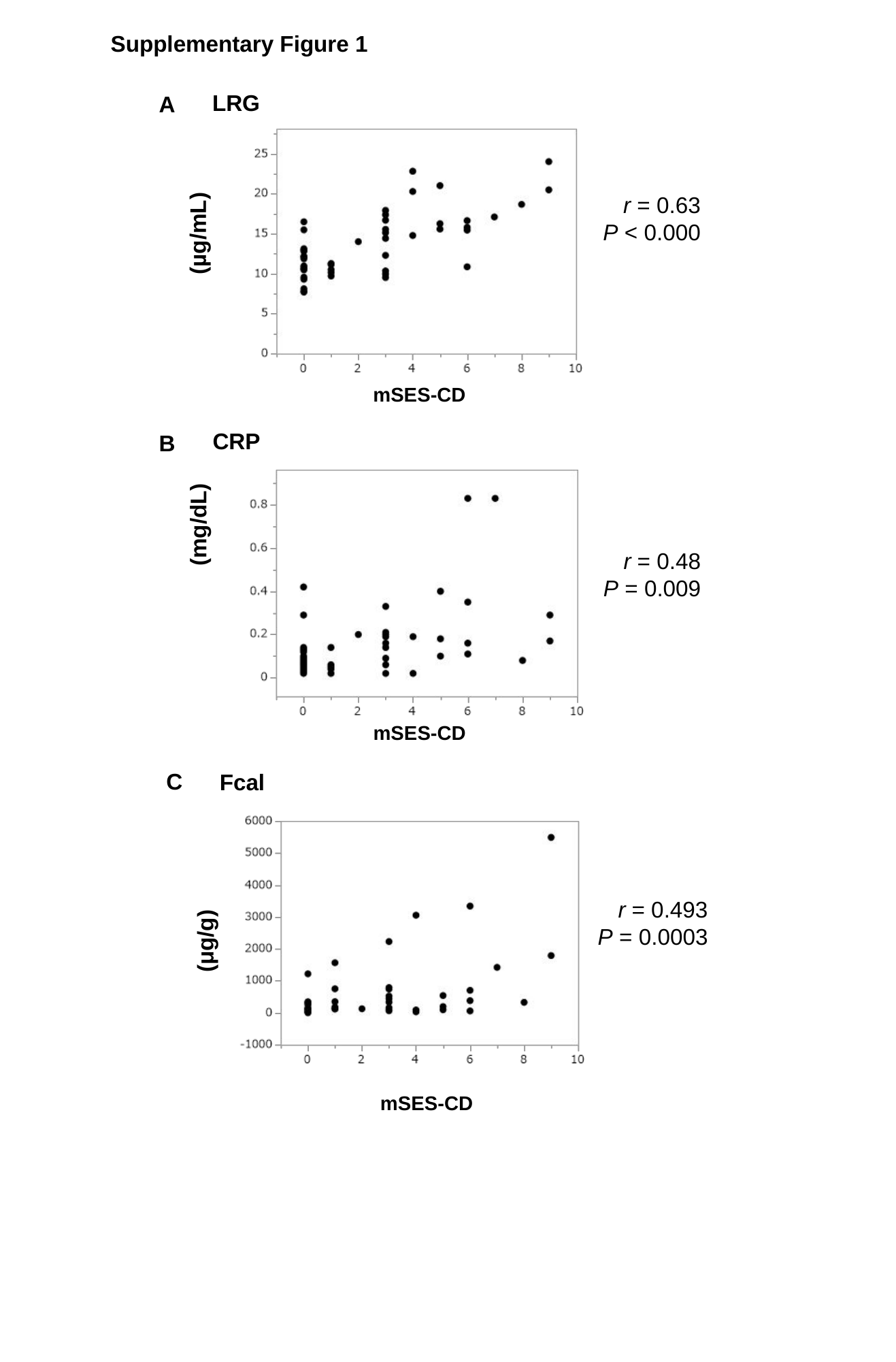

Supplementary Figure 1
LRG
A
r = 0.63
P < 0.000
(µg/mL)
mSES-CD
CRP
B
(mg/dL)
r = 0.48
P = 0.009
mSES-CD
C
Fcal
r = 0.493
P = 0.0003
(μg/g)
mSES-CD

## Slide 4
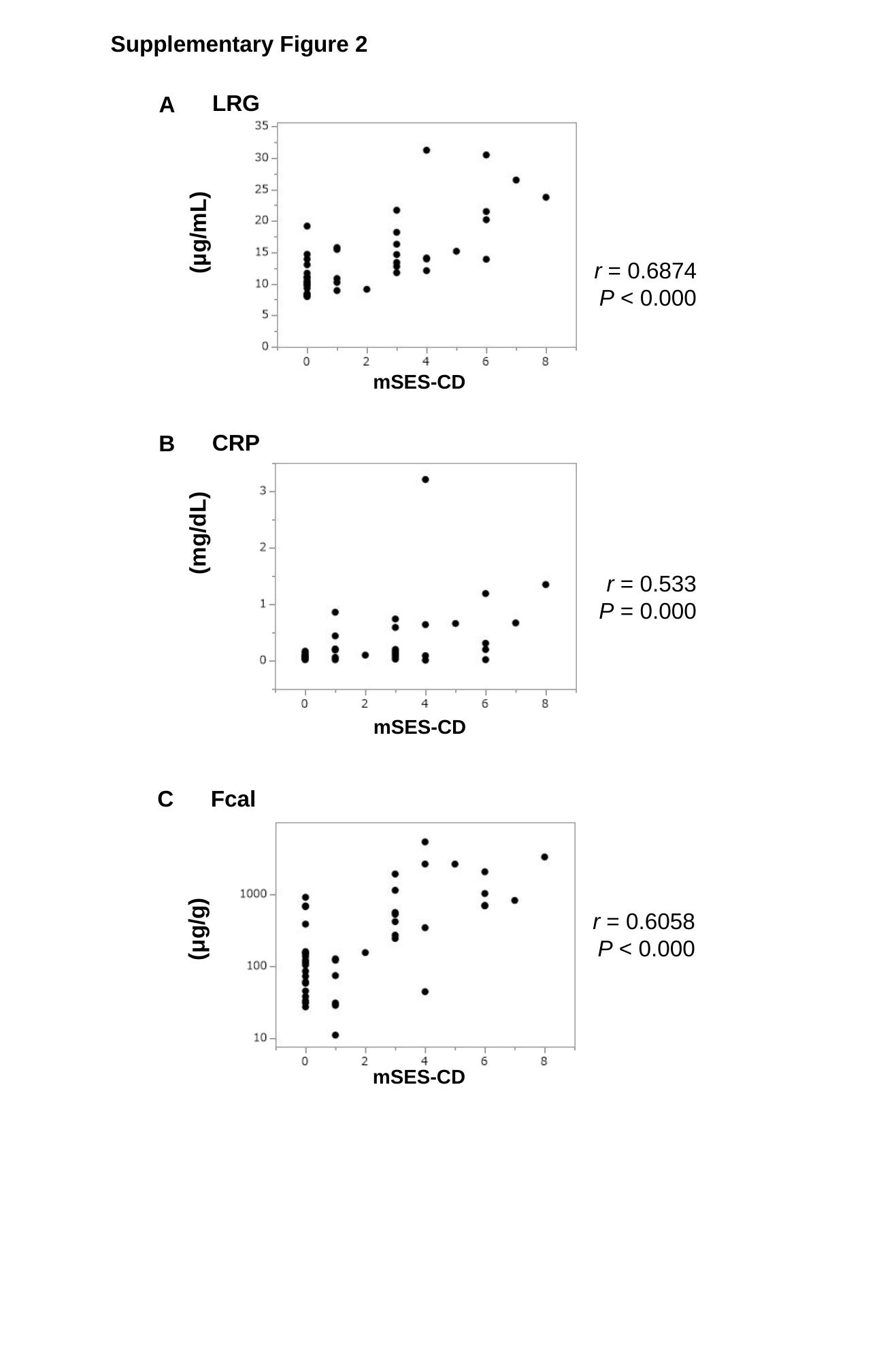

Supplementary Figure 2
LRG
A
(µg/mL)
r = 0.6874
P < 0.000
mSES-CD
CRP
B
(mg/dL)
r = 0.533
P = 0.000
mSES-CD
C
Fcal
(μg/g)
r = 0.6058
P < 0.000
mSES-CD

## Slide 5
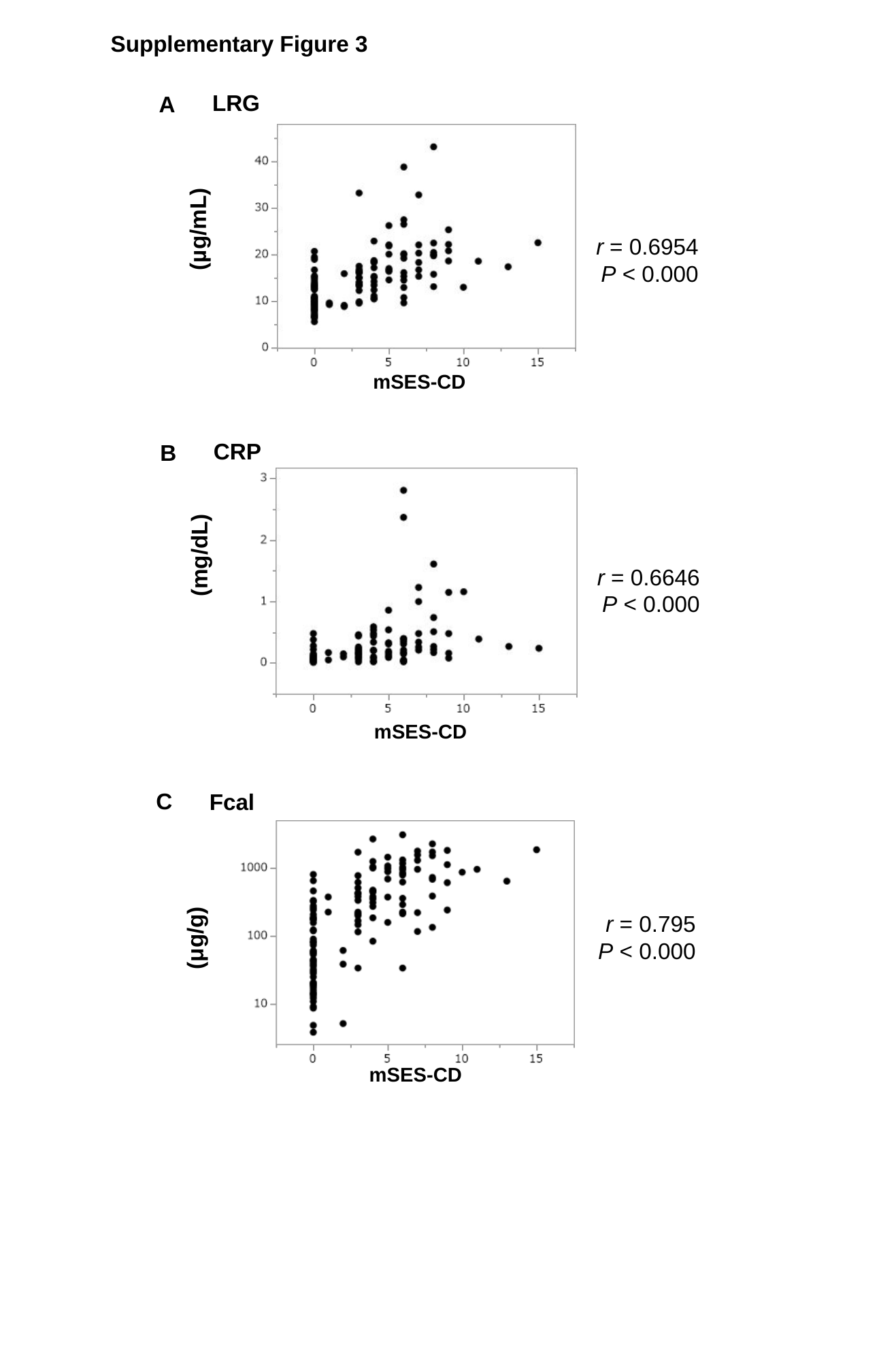

Supplementary Figure 3
LRG
A
(µg/mL)
r = 0.6954
P < 0.000
mSES-CD
CRP
B
(mg/dL)
r = 0.6646
P < 0.000
mSES-CD
C
Fcal
(μg/g)
r = 0.795
P < 0.000
mSES-CD

## Slide 6
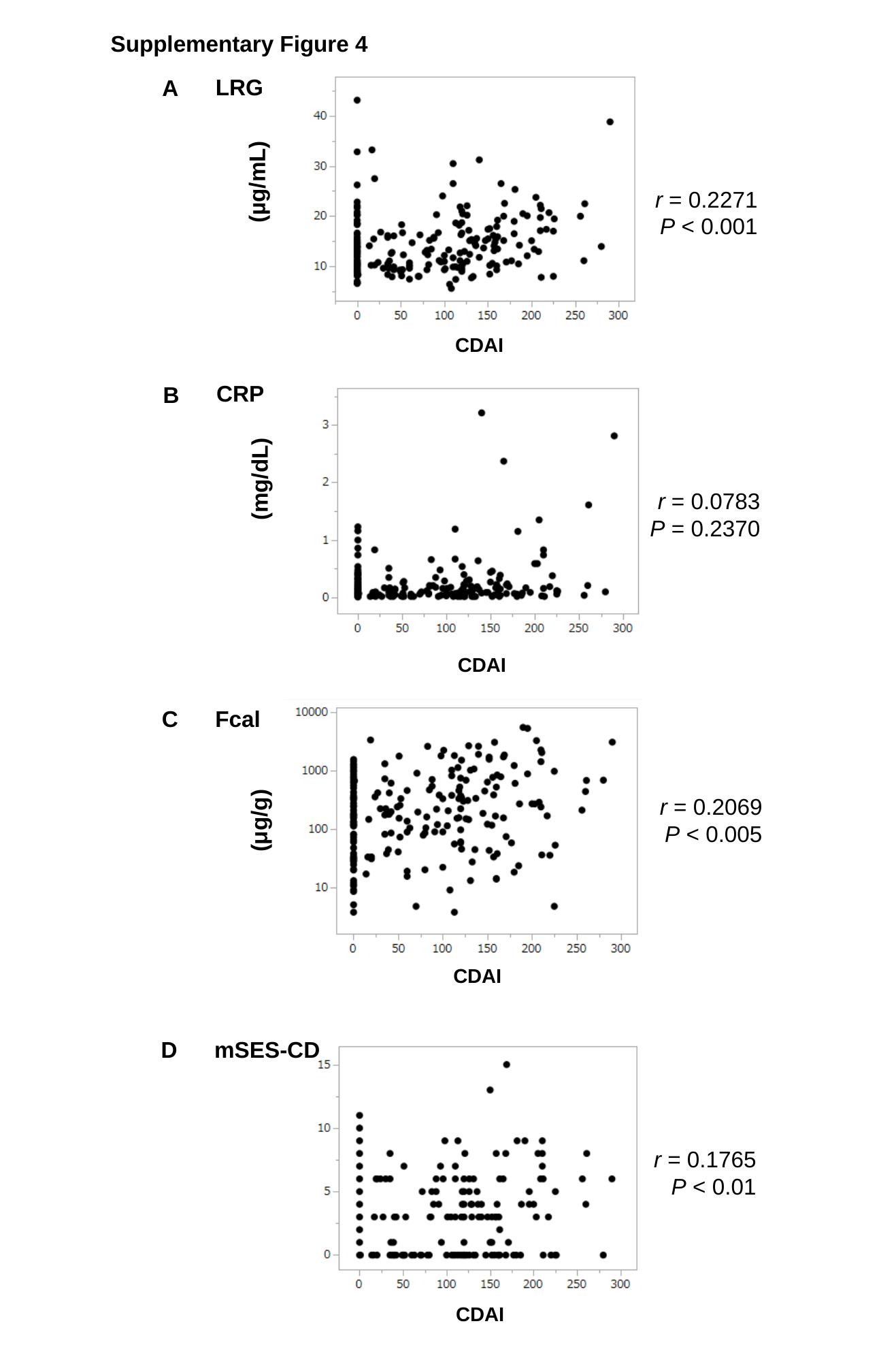

Supplementary Figure 4
LRG
A
(µg/mL)
r = 0.2271
P < 0.001
CDAI
CRP
B
(mg/dL)
r = 0.0783
P = 0.2370
CDAI
C
Fcal
(μg/g)
r = 0.2069
P < 0.005
CDAI
D
mSES-CD
r = 0.1765
P < 0.01
CDAI
