# Supplementary material for: Efficient diagnosis for endoscopic remission in Crohn’s diseases by the combination of three non-invasive markers
Source: BMC Gastroenterol. 2025 May 13;25:364. doi: 10.1186/s12876-025-03880-5 (PMC12070669; doi:10.1186/s12876-025-03880-5)
Supplement: Supplementary file 2 — Supplementary Material 2. [file 12876_2025_3880_MOESM2_ESM.docx]

**Supplementary Table 1. Diagnostic ability of endoscopic remission based on different cut-offs for fecal calprotectin and CRP recommended by AGA**

| Cut-off values derived from the ROC of this study | | | | | |
| --- | --- | --- | --- | --- | --- |
|  | Sensitivity (95% CI) | Specificity (95% CI) | PPV (95% CI) | NPV (95% CI) | Accuracy (95% CI) |
| Fcal < 180 μg/g | 79.8%  (72.6-87.0) | 82.9%  (75.9-89.9) | 83.3%  (76.5-90.2) | 79.3%  (71.9-86.7) | 81.3%  (76.3-86.3) |
| CRP < 0.15 mg/dL | 84.2%  (76.9-91.5) | 74.8%  (67.5-82.1) | 70.2%  (61.8-78.6) | 87.1%  (81.0-93.2) | 78.7%  (73.4-84.0) |
| Cut-off values recommended by AGA | | | | | |
|  | Sensitivity (95% CI) | Specificity (95% CI) | PPV (95% CI) | NPV (95% CI) | Accuracy (95% CI) |
| Fcal < 50 μg/g | 59.2%  (51.8-66.6) | 92.2%  (84.8-99.5) | 96.2%  (92.5-99.8) | 40.5%  (31.6-49.5) | 66.8%  (60.6-73.0) |
| Fcal < 150 μg/g | 73.0%  (65.6-80.4) | 84.9%  (77.7-92.2) | 87.7%  (81.7-93.7) | 68.1%  (59.6-76.6) | 77.8%  (72.5-83.2) |
| Fcal < 250 μg/g | 81.1%  (73.7-88.6) | 77.4%  (70.1-84.8) | 75.4%  (67.5-83.3) | 82.8%  (75.9-89.6) | 79.1%  (73.9-84.4) |
| CRP < 0.50 mg/dL | 93.1%  (83.9-1.00) | 56.7%  (49.9-63.6) | 23.7%  (15.9-31.5) | 98.3%  (95.9-1.00) | 61.3%  (55.0-67.6) |

PPV, positive predictive value; NPV, negative predictive value; CRP, C-reactive protein; Fcal, fecal calprotectin; CI, confidence interval.
